# Secondary hemophagocytic lymphohistiocytosis in the setting of metastatic renal cell carcinoma: a case report

**DOI:** 10.1186/s13256-016-1196-9

**Published:** 2017-03-02

**Authors:** Monica El-Masry, Lauren Eisenbud, Minh-Ha Tran

**Affiliations:** 1 Department of Internal Medicine, UC Irvine Health School of Medicine, 101 The City Drive S, Orange, CA 92868 USA; 2 Department of Internal Medicine, Arrowhead Regional Medical Center, 400 N Pepper Avenue, Colton, CA 92324 USA; 3 Department of Pathology and Laboratory Medicine, UC Irvine Health School of Medicine, 101 The City Drive S, Orange, CA 92868 USA

**Keywords:** Hemophagocytic lymphohistiocytosis, Disseminated intravascular coagulation, Fever, Fever of unknown origin, Altered mental status

## Abstract

**Background:**

Hemophagocytic lymphohistiocytosis is a disease process characterized by unregulated hyperactivation of the immune system associated with multiorgan involvement and high mortality rates. Early recognition is crucial and a recently validated diagnostic schema, the H-Score, may facilitate diagnosis particularly in secondary hemophagocytic lymphohistiocytosis cases. We present a patient with secondary hemophagocytic lymphohistiocytosis in association with metastatic renal cell carcinoma in whom high-dose steroid therapy induced a remarkable response.

**Case presentation:**

A 35-year-old Vietnamese man with quiescent systemic lupus erythematosus was diagnosed 5 months prior to admission with left-sided renal cell carcinoma metastatic to the pancreas and spine. Ten days prior to admission, a febrile illness (temperatures to 39 °C) associated with flu-like symptoms unresponsive to levofloxacin developed. He took only two doses of pazopanib prior to admission. High fevers unresponsive to antimicrobial therapy, cytopenias, disseminated intravascular coagulation, and progressive multiorgan failure led to intubation and intensive care unit stay. Extensive infectious disease workup showed only negative results, but elevation of interleukin-2 receptor, exceedingly high ferritin levels and other features earned an H-Score of 302, consistent with >99% diagnostic probability for secondary hemophagocytic lymphohistiocytosis. High-dose steroid therapy produced a rapid clinical and biochemical response.

**Conclusions:**

Hemophagocytic lymphohistiocytosis is a life-threatening disorder which is likely to be under-recognized. Increased awareness of this disease entity and its diagnosis is crucial toward early recognition and treatment. To our knowledge, our patient is only the second reported with secondary hemophagocytic lymphohistiocytosis occurring in the setting of renal cell carcinoma.

## Background

Hemophagocytic lymphohistiocytosis (HLH) is a disease process characterized by unregulated hyperactivation of the immune system resulting in variable combinations of fever, multiorgan involvement (particularly transaminase elevation), and peripheral blood cytopenias [1]. Presentations are generally divided into primary and secondary forms, the former typically occurring in infants or young children and often incorporating either autosomal recessive mutations in any of several recognized familial hemophagocytic lymphohistocytosis (FHL) genes, or as a feature of other, non-FHL immunodeficiency syndromes (see Table 1) [2, 3]. Primary HLH, which has an incidence of 1.2 per million children per year [4], is diagnosed based upon criteria set forth by the Histiocyte Society [5] (see Table 1).

**Table 1:** Hemophagocytic lymphohistiocytosis (HLH) 2004 diagnostic criteria (Adapted with permission from John Wiley and Sons Ltd. from Brisse *et al*. [3] and Henter *et al*. [5]). Diagnostic criteria 1–5 represent the original 1991 diagnostic criteria; the HLH 2004 revision adds criteria 6–8. The diagnosis of primary HLH can be made either on a molecular or clinical basis

HLH Molecular diagnosis		At least 5 of the following 8 diagnostic criteria	
FHL genes	*PRF1*, *UNC13D*, *STX11*, *STXBP2*	1. Fever	2. Splenomegaly
		3. Cytopenias (≥2/3 lineages)^a^	4. High triglyceride/low fibrinogen levels^b^
Non-FHL genes	*RAB27A*, *LYST*, *AP3B1*, *SH2D1A*, *XIAP*	5. Hemophagocytosis	6. Low/absent NK-cell activity
		7. Ferritin ≥500 ng/mL	8. Soluble IL-2 receptor ≥2400 U/mL

*FHL* Familial hemophagocytic lymphohistocytosis, *NK* Natural Killer, and *IL* Interleukin. non-FHL gene mutations occur in Gricselli syndrome, Chediak-Higashi syndrome, Hermansky-Pudlak syndrome type 2, and X-linked lymphoproliferative disease

^a^Hb <9 g/dL, platelets <100 K/mcL, neutrophils <1.0 K/mcL. ^b^Triglycerides ≥265 mg/dL, fibrinogen ≤150 mg/dL

Secondary HLH occurs typically in adolescents or adults and in the setting of infectious, rheumatologic (i.e., juvenile idiopathic inflammatory arthritis), or malignant conditions (i.e., lymphoma) [6]. The HLH-2004 criteria may have reduced sensitivity and specificity in secondary HLH - where sufficient criteria may not be met at disease onset but develop later in the course and associated malignancy and inflammatory conditions may predispose to higher baseline ferritin values [3]. It has, therefore, been suggested that this disorder is likely under-recognized in modern practice [7]. Treatment is largely based upon the original HLH-94 protocol [8] incorporating an initial 8-week induction regimen using corticosteroids in most patients and chemotherapy (etoposide) in selected patients (i.e., when Epstein-Barr virus (EBV)-driven disease is present). Despite treatment, the mortality of secondary HLH ranges from 32.4% to as high as 87.5% [9, 10].

We recently encountered a 35-year-old male patient with a history of systemic lupus erythematosus (SLE) and metastatic renal cell carcinoma, who presented with a flu-like illness. Fevers, multiorgan failure, altered mental status, cytopenias, and disseminated intravascular coagulation (DIC) findings progressed rapidly in the face of empiric antimicrobial and antiviral therapy. Diagnosis of secondary HLH and immediate institution of corticosteroid therapy was associated with a dramatic clinical response.

## Case presentation

A 35-year-old Vietnamese man with underlying SLE who was taking hydroxychloroquine, azathioprine, and prednisone was diagnosed 5 months prior to admission with metastatic renal cell carcinoma with a 6.6 × 6.0 × 7.2-cm left renal mass with disruption of fat planes, suggestive of involvement of the tail of the pancreas, as well as a 4.1 × 3.2 × 2.6-cm mass at the T8 spinal vertebra. On hospital day (HD) -43, he underwent hemivertebrectomy with fusion of adjacent vertebral levels; pathologic examination of the T8-mass demonstrated renal cell carcinoma. He was first evaluated at our institution for a second opinion for his malignancy on HD −21. Plans were put forth to obtain outside records and undertake multidisciplinary treatment planning during tumor board.

However, on approximately HD −10, he developed a febrile illness (temperatures to 38.9 °C measured at home) with flu-like symptoms comprised of shortness of breath, frontal headaches, sore throat with neck pain, nausea, vomiting, diarrhea, and generalized weakness. He took pazopanib 800 mg daily, which had been prescribed prior to onset of illness, on HDs −3 and −2. Despite an outpatient course of levofloxacin, progression of symptoms led ultimately to presentation on HD 0.

Presenting vital signs in the emergency department included fever of 39.2 °C, tachycardia of 101 beats per minute (bpm), and relative hypotension at 106/66 mmHg. Initial laboratory studies demonstrated multiorgan involvement with pancytopenia, acute kidney injury, hyponatremia, abnormal liver function tests, and elevations in lactic acid, procalcitonin, and lipase levels. Clinically, he was judged to be euvolemic. Further studies noted that serum osmolality was low at 267 mOsm/kg, spot urine sodium 24 mmol/L, and urine osmolality 776 mOsm/kg. A plain film of the chest was normal, save for spinal hardware, and cranial computed tomography showed no acute intracranial process. His SLE was clinically judged to be quiescent, corroborated by an anti-double-stranded deoxyribonucleic acid (anti-dsDNA) antibody screen and an extensive infectious disease workup both of which showed only negative results (see Table 2).

**Table 2:** Results of infectious disease workup

Source	Test result						
Blood	Cultures: no growth (bacterial/fungal)	Negative for HIV 1/2 and HIV p24 Ag	Nonreactive for Lyme antibody and *Treponema pallidum* immunoassay	Negative for *Coccidioides* IgM and IgG. Negative for *Histoplasma* Ag	Viral hepatitis panel (A, B, C) significant only for hepatitis B core Ab	EBV serology consistent with seropositive status^a^	CMV serology consistent with seronegative status
Urine	Urinalysis significant only for trace proteinuria	Negative for *Legionella* Ag	Negative for *Streptococcus pneumoniae* Ag				
Nasopharyngeal swab	Negative for Group A Streptococcus (direct antigen test)	Negative for influenza A, B, RSV (rt-PCR)	Negative for MSSA and MRSA				
Cerebrospinal fluid (CSF)	CSF showed 14 nucleated cells/mm^3^ (normal 0–5/mm^3^) consisting of 84% polymorphonuclear leukocytes, 9% lymphocytes, and 7% monocytes; 44 mg/dL glucose (normal 41–70 mg/dL); 70 mg/dL protein (normal 15–45 mg/dL)			*Cryptococcus neoformans* Ag and *C. gattii* Ag	Nonreactive for West Nile virus IgG/IgM	Negative for HSV 1, 2 and enterovirus (PCR)	Negative for acid fast bacilli, no growth occurred during fungal and mycobacterial culture
Stool	Negative for *Clostridium difficile* toxin B (PCR)						
Other	PPD skin test 0-mm induration	Quantiferon Gold tuberculosis test indeterminate					

*Ab* antibody, *Ag* antigen, *CMV* cytomegalovirus, *EBV* Epstein-Barr virus, *HSV* herpes simplex virus, *Ig* immunoglobulin, *MSSA* methicillin-sensitive *Staphylococcus aureus*, *MRSA* methicillin-resistant *Staphylococcus aureus, PCR* polymerase chain reaction, *PPD* purified protein derivative, *RSV* respiratory syncytial virus

^a^EBV viral capsid, nuclear, and early diffuse IgG Abs reactive, viral capsid IgM nonreactive; EBV PCR not performed

He was started on broad-spectrum antibiotics (vancomycin and piperacillin-tazobactam) and admitted to the internal medicine service. On HD 2, given a declining mental status and concern for meningitis, piperacillin-tazobactam was discontinued, and ceftriaxone, acyclovir, and ampicillin were added to the vancomycin. Ultimately, the patient required intubation for airway protection and was transferred to the medical intensive care unit (ICU). Lumbar puncture was performed and the results are presented in Table 2.

On HD 2 he developed urinary retention with a 600-ml output following straight catheterization prompting placement of a Foley catheter. Also on HD 2, loose stools developed along with increased abdominal rigidity and a rise in lactic acid levels from an admission value of 1.8 mmol/L (normal 0.5–2.2) to 4.9 mmol/L. Stool polymerase chain reaction (PCR) was negative for *Clostridium difficile* toxin B. His mental status deteriorated and he became agitated and required intubation on HD 2 for airway protection and was transferred to the medical ICU.

Laboratory values on HD 2 included elevated aspartate aminotransferase (AST, normal range 13-39 U/L)/alanine aminotransferase (ALT, normal range 7-52 U/L) of 547 and 118 U/L, respectively; declining albumin from 2.7 to 1.7 mg/dL; and elevated LDH of 2833 U/L (normal 140-271 U/L), lipase of 501 U/L (normal 11–82 U/L), and triglycerides of 500 mg/dL (normal <150 mg/dL). Iron studies revealed a low transferrin of 99.3 mg/dL (normal 203–362 mg/dL) and TIBC of 139 mcg/dL (284–507 mcg/dL), low-normal serum iron of 50 mcg/dL (normal 49–181 mcg/dL), and normal saturation of 36% (normal 20–55%). Ferritin was substantially elevated at >7500 ng/mL (normal 23–233 ng/mL) and later peaked at >15,000 ng/mL.

The Quantiferon Gold tuberculosis (TB) test resulted as indeterminate due to a high degree of nonspecific reactivity produced by the patient’s specimen (i.e., NIL (negative control tube) >10 IU/mL). This was in concert with a very high C-reactive protein (CRP) result of 25.9 mg/dL (normal range 0.0–1.0 mg/dL) and an elevated soluble interleukin (IL)-2 receptor result of 1112 pg/mL (normal ≤1033 pg/mL). A purified protein derivative (PPD) skin test yielded 0-mm induration.

Despite broad-spectrum antibiotics, the patient remained persistently febrile, with daily fevers exceeding 38.4 °C (maximal temperature was on HD 2 at 38.9 °C). He developed rigors and a subsequent mild rhabdomyolysis with creatinine kinase values increasing to a maximum of 7546 U/L (normal 30–223 U/L) on HD 2. Aggressive cooling measures were required, including acetaminophen and a cooling blanket. Continuous rigors with abdominal rigidity precluded accurate physical examination for hepatosplenomegaly. A kidneys-ureters-bladder (KUB) plain film taken on HD 2, however, had findings consistent with possible hepatosplenomegaly.

A coagulopathy developed with maximal values on HD 5 of Prothrombin Time (PT) 29.7 s (normal 11.5–14.1 s), International Normalized Ratio (INR) of 2.91 (0.87–1.13), Partial Thromboplastin Time (PTT) 63.5 s (normal 24.7–37.0 s), and nadir fibrinogen level of 76 mg/dL (211–410 mg/dL). The latter prompted cryoprecipitate transfusions on HD 4 and HD 6. Progression of coagulopathy occurred in parallel with progression of thrombocytopenia, with nadir platelet count by HD 9 of 25 K/mcL (normal 150–400 K/mcL). Nadir hemoglobin also occurred on HD 9 at 7 g/dL. Schistocytosis was absent and neither red blood cell nor platelet transfusions were required throughout the hospital stay.

The combination of fever (maximal recorded 39.2 °C) in the setting of an infectious disease workup which showed only negative results, with trilineage cytopenias, elevated AST and ALT, hypofibrinogenemia (nadir of 76 mg/dL), significant hyperferritinemia (peak >15,000 ng/mL), triglycerides of 500 mg/dL, as well as suggestion on KUB imaging for hepatosplenomegaly, was compatible with a secondary HLH diagnosis. Involvement of the central nervous system (CNS) was evident given his mental status changes and agitation. Additionally, natural killer (NK)-cell function was reduced with a Lytic Unit 30 value of 5 lytic sets (normal LU30 = 7–125 lytic sets) and soluble IL-2 receptor level modestly elevated at 1112 pg/mL (normal ≤1033).

**Fig. 1:**
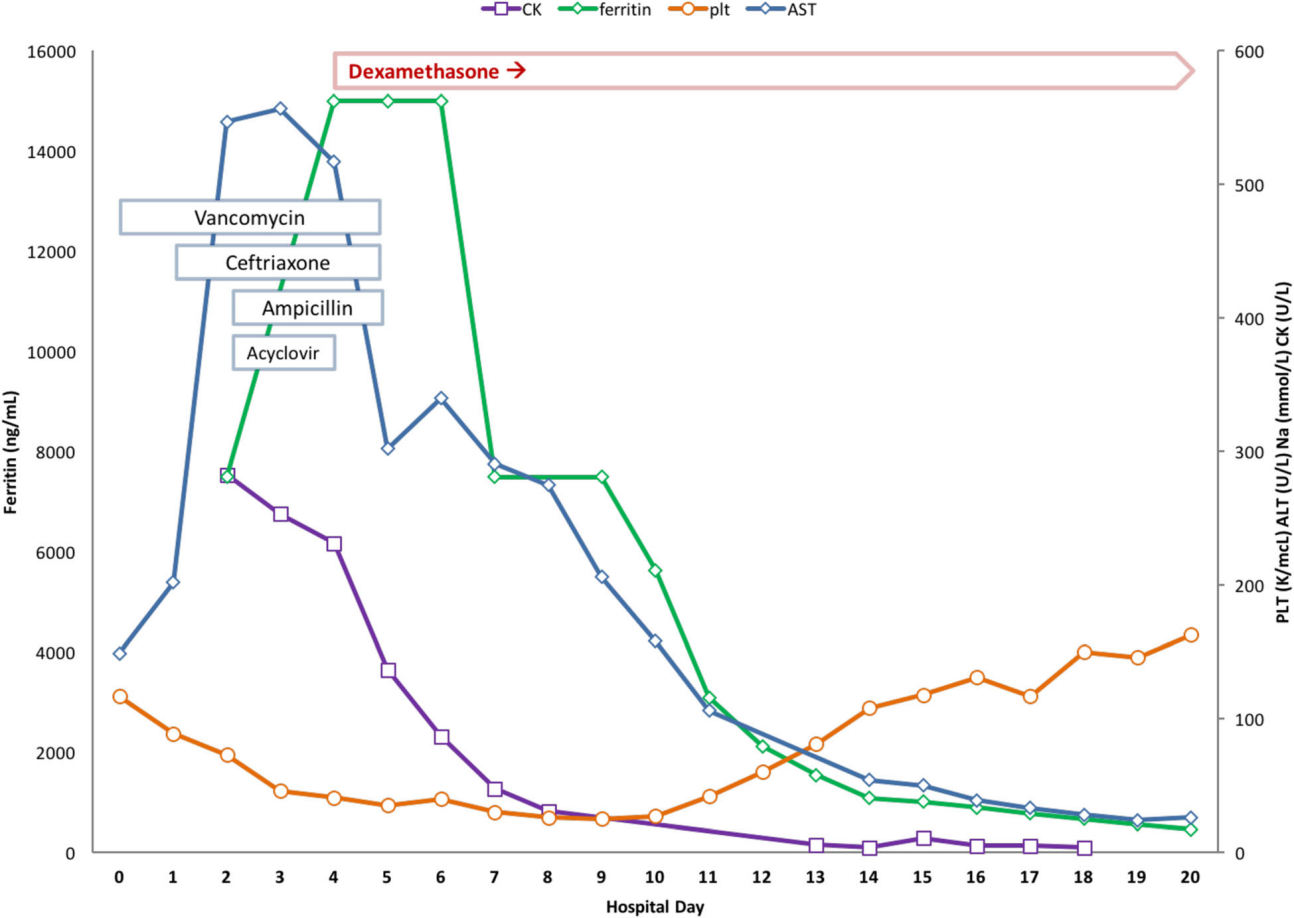
Timeline. Improvement in laboratory values with initiation of dexamethasone. *PLT* platelet count (normal 150–400 K/mcL), *AST* aspartate aminotransferase (normal 13–39 U/L), *CK* creatinine kinase (normal 30–223 U/L)

High-dose steroids were, therefore, initiated. Dexamethasone, preferred because it can cross the blood-brain barrier, was initiated at 10 mg/m^2^ daily for weeks 1 and 2, followed by 5 mg/m^2^ daily for weeks 3 and 4, then 2.5 mg/m^2^ daily for weeks 5 and 6, then 1.25 mg/m^2^ daily for week 7 with tapering to zero during week 8. Clinical response was immediately noted with resolution of fevers and rigors and rapidly improving mental status. Laboratory abnormalities also improved; their trends following initiation of dexamethasone are further detailed in Fig. 1. Our patient was subsequently extubated on HD 5 and discharged in ambulatory condition with full cognitive ability on HD 20.

## Discussion

Our 35-year-old patient developed secondary HLH in the setting of preexisting immunosuppression due to underlying SLE and metastatic renal cell carcinoma. To our knowledge, this is the first case of HLH reported in the setting of pazopanib therapy and only the second case reported in association with renal cell carcinoma (RCC) [11].

Based upon a validated cohort of secondary HLH patients [12], Fardet *et al*. developed a diagnostic H-Score [13] to predict the likelihood of secondary HLH. Points are assigned based upon presence of:

- Three clinical features
  - ○ Known immunosuppression – no: 0/yes: 18; high temperature <38.4 °C: 0, 38.4 to 39.4 °C: 33, >39.4 °C: 49; neither hepatomegaly nor splenomegaly: 0, either: 23, or both: 38
- Five biologic features
  - ○ Triglycerides mg/dL <132.9: 0, 132.9 to 354.3: 44, >354.3: 64; ferritin ng/mL <2000: 0, 2000–6000: 35, >6000: 50; AST U/L <30: 0, ≥30: 19; fibrinogen mg/dL >250: 0, ≤250: 30; number of cytopenic lineages – 1: 0, 2: 24, 3: 34
- One cytologic feature
  - ○ Hemophagocytosis – no: 0, yes: 35

In regards to the validation cohort, the median (IQR) H-Score in the group with and without secondary HLH was 230 (203–257) and 125 (91–150), respectively. At H-Scores of 90, 150, 190, 200, 220, 230, 240, and 250, the probability for secondary HLH was <1%, 25%, 80%, 88%, 96%, 98%, 99%, and >99%, respectively [13]. Performance of the H-Score was compared against that of the adapted HLH-2004 criteria in a validation study by Debaugnies *et al*. [14]. In their comparison, the HLH-2004 criteria were shortened to exclude low NK-cell activity and soluble IL-2 receptor levels, thereby having six total criteria instead of the original eight. Among adult patients at initial presentation, the H-Score outperformed the modified HLH-2004 criteria at disease recognition: an H-Score of >138 offered 90% sensitivity, 79% specificity, and 86% diagnostic accuracy compared to the scenario in which five out of six HLH-2004 criteria are met, where respective values were: 55%, 100%, and 80%. For established disease, and using maximal score values, the two diagnostic criteria (using an H-Score >185) performed similarly.

In addition to meeting at least five out of the eight criteria in the original HLH-2004 diagnostic guidelines [5], our patient also had a diagnostic H-Score. Qualifying elements included: known immunosuppression (SLE on hydroxychloroquine, azathioprine, and steroids), a maximum temperature of 39.6 °C, hepatosplenomegaly, triglycerides of 500 mg/dL, AST >30 U/L, ferritin >6000 ng/mL, fibrinogen nadir <250 mg/dL, and trilineage cytopenias, earning an H-Score of 302, consistent with a diagnostic probability of >99%. The dramatic improvement on all fronts that ensued following initiation of steroid therapy lends further confidence to accuracy of our diagnosis.

Other features consistent with secondary HLH in our patient include the presence of a euvolemic, hypo-osmolar hyponatremia with spot urine sodium of 24 mmol/L, consistent with the syndrome of inappropriate antidiuretic hormone (SIADH) [1]. Additionally, our patient manifested CNS involvement with encephalopathy and agitation requiring intubation for airway protection.

The most common viral drivers for secondary HLH are EBV, HIV, herpes viruses, cytomegalovirus (CMV), viral hepatitis, and influenza [1]. In our patient, viral-testing was negative for these entities. Bacterial, fungal, and mycobacterial cultures also tested negative. SLE is also a recognized driver [1], but our patient did not appear to have signs of active disease, suggesting that the HLH was secondary to metastatic renal cell carcinoma.

## Conclusions

HLH is a life-threatening disorder resulting from immunologic hyperactivation that can progress to multiorgan failure. Early recognition is critical so that progression may be interrupted. However, this disease entity is likely under-recognized which is of concern given its high mortality rate. In pediatric cases, diagnosis is based upon the HLH-2004 criteria; which, for adult cases, may be insensitive for diagnosis earlier in the disease course. The H-Score is a validated diagnostic tool more appropriate for use among secondary HLH cases. For pediatric or EBV/lymphomatous cases, chemotherapeutic agents are often added. For adult cases, a high-dose dexamethasone regimen based upon the HLH-94 protocol may be used.
